# Brain-Derived Cystathionine β-Synthase-Generated H_2_S Attenuates Cerebral Ischemia–Reperfusion Injury via VEGFR_2_-Mediated Angiogenesis in MCAO/R Rats

**DOI:** 10.3390/cimb48040418

**Published:** 2026-04-18

**Authors:** Shuai Liang, La Jiang, Yu Jiang, Shan Wang, Jia-Rong Jiang, Ji-Yue Wen, Zhi-Wu Chen, Shuo Chen

**Affiliations:** 1 Department of Pharmacology, School of Pharmaceutical Sciences, Anhui Medical University, Hefei 230032, China; 2345010187@stu.ahmu.edu.cn (S.L.); 2346010032@stu.ahmu.edu.cn (L.J.); wenjiyue139@aliyun.com (J.-Y.W.); 2 Clinical Medical College, Anhui Medical University, Hefei 230032, China; 3 Key Laboratory of Xin’an Medicine, Ministry of Education, Anhui University of Chinese Medicine, Hefei 230038, China

**Keywords:** ischemic stroke, cystathionine β-synthase, H_2_S, VEGFR_2_, angiogenesis

## Abstract

Ischemic stroke (IS) remains a major cause of global disability and mortality. While exogenous H_2_S has demonstrated neuroprotective potential, the role of endogenous H_2_S generated by cystathionine β-synthase (CBS) in cerebral ischemia–reperfusion injury (CIRI) remains incompletely elucidated. L-Cysteine (L-Cys), as a substrate for CBS, serves as a key precursor for endogenous H_2_S. Using the established pre-clinical model of CIRI—middle cerebral artery occlusion/reperfusion (MCAO/R) in rats—we investigated the neuroprotective effects of brain-derived CBS-generated H_2_S through neurological function scoring, 2,3,5-triphenylchlorotetrazole (TTC) staining, enzyme-linked immunosorbent assay (ELISA), and histopathological examination. Immunofluorescence, Western blot, and laser speckle contrast imaging were utilized to analyze the protein expression of ZO-1, claudin-5, CBS, vascular endothelial growth factor receptor-2 (VEGFR_2_) and CD31, as well as cerebral blood flux changes. L-Cys treatment ameliorated neurological deficits, reduced cerebral infarct volume, decreased serum lactate dehydrogenase (LDH) and neuron-specific enolase (NSE) levels, attenuated histopathological damage, alleviated cerebral edema, and restored blood–brain barrier integrity via upregulation of tight junction proteins ZO-1 and claudin-5. Additionally, L-Cys improved MCAO/R-induced cognitive impairment and behavioral deficits. Furthermore, L-Cys upregulated CBS and VEGFR_2_ expression, enhanced endogenous H_2_S production, promoted post-ischemic cerebral angiogenesis, and improved cerebral blood flux recovery. CBS-derived H_2_S promoted post-ischemic angiogenesis mediated by VEGFR_2_, enhances cerebral reperfusion flux, and consequently ameliorated MCAO/R-induced CIRI in rats, providing experimental evidence for clinical translation.

## 1. Introduction

Stroke is recognized worldwide as a major cause of death and disability. This disease is primarily caused by severe damage to brain tissue resulting from ruptured blood vessels or vascular blockages. In 2021, ischemic stroke (IS) accounted for 65.3% of all new stroke cases worldwide (approximately 7.804 million cases), resulting in 3.591 million deaths [1]. Currently, the most common treatment for clinical patients with acute ischemic stroke (AIS) is endovascular therapy (EVT) via intravenous thrombolysis or arterial thrombectomy. While EVT has dramatically improved complete reperfusion rates in AIS patients, its clinical use remains limited due to a narrow treatment window, strict contraindications, and the risk of hemorrhagic transformation. A substantial proportion of patients (27.6–56.7%) experience poor functional outcomes despite successful recanalization—a phenomenon termed ‘futile reperfusion’ [2,3,4]. Moreover, patients who achieve successful vascular recanalization remain susceptible to CIRI. Therefore, strategies to enhance effective tissue reperfusion and mitigate ischemia–reperfusion injury are of paramount importance.

Since NO was identified as an endothelium-derived relaxing factor in the 1980s, its pivotal role as the first discovered gaseous signaling molecule in the cardiovascular, nervous, and immune systems has been extensively elucidated [5]. Subsequently, CO was also found to possess similar signaling functions [6]. H_2_S was recognized for its toxicity and environmental hazards. However, recent research has established its physiological significance as a gaseous neurotransmitter involved in diverse physiological and pathological processes [7]. In IS, H_2_S has been shown to mitigate oxidative stress, neuroinflammation and apoptotic pathways. Furthermore, it is now recognized as a gaseous transmitter that regulates angiogenesis under both physiological and ischemic conditions, conferring vascular protective effects in pathological states [8]. Endogenous H_2_S production in mammals is primarily catalyzed by three enzymes: cystathionine-γ-lyase (CSE), CBS, and 3-mercaptopyruvate sulfurtransferase (3-MST) [9]. CBS is essential for endogenous H_2_S synthesis in the brain [10]. L-Cys, as a substrate for CBS, serves as a key precursor for brain-derived H_2_S [11]. The CBS-catalyzed H_2_S production pathway utilizes L-Cys and L-homocysteine as dual substrates, generating L-cystathionine while releasing H_2_S. L-cystathionine itself is not a metabolic end product but a key intermediate in the transsulfuration pathway—it is converted back to L-Cys under the catalysis of CSE [12]. The pro-angiogenic effects of either direct H_2_S inhalation or H_2_S donor administration have been extensively validated across multiple ischemic models [13,14]. However, it remains unclear whether brain-derived H_2_S exerts protective effects in IS.

Angiogenesis constitutes a critical component of neurovascular remodeling post-stroke, wherein new capillaries form through the targeted proliferation and migration of endothelial progenitor cells from pre-existing vasculature [15]. Angiogenesis can be observed within days following IS, with higher microvessel density correlating with improved patient survival outcomes [16]. Enhanced angiogenesis promotes collateral circulation, restoring oxygen and energy supply to ischemic tissue while providing neurotrophic support [10] for neurogenesis and synaptogenesis—processes that collectively contribute to functional recovery [17,18]. VEGFR_2_ mediates the majority of VEGF’s pro-angiogenic effects. Activation of VEGFR_2_ inhibits endothelial cell apoptosis, enhances cellular viability, and stimulates proliferation, migration and tube formation—critical steps in angiogenesis [19]. Research by Tao et al. has shown that H_2_S is capable of cleaving the disulphide bond between Cys1045 and Cys1024 in VEGFR_2_, thereby restoring the active conformation of VEGFR_2_ [20]. Our previous studies have also demonstrated that H_2_S binds specifically to VEGFR_2_ [21], and that NaHS significantly enhances the phosphorylation of VEGFR_2_ at the Tyr797 and Ser799 sites. This is considered to be key to the protective effect of NaHS against hypoxia–reoxygenation injury in rat brain artery endothelial cells [22]. Therefore, this study aims to investigate the therapeutic potential of brain-derived H_2_S in MCAO/R rats, focusing on elucidating its role in promoting angiogenesis and enhancing effective cerebral reperfusion flux through the VEGFR_2_ signaling pathway, thereby providing experimental and theoretical foundations for its clinical application in IS treatment.

## 2. Materials and Methods

### 2.1. Materials and Reagents

The following reagents were commercially obtained: MCAO filament (L3600, Jialing Biotech, Guangzhou, China); L-Cys (C16152035, Macklin, Shanghai, China); VEGF164 (CJ96, Novoprotein, Suzhou, China); AOAA (Aminooxyacetic acid, MX5314, Maokang Bio, Shanghai, China); SU5416 (Semaxinib, HY-10374, MedChemExpress, Monmouth Junction, NJ, USA); LDH (A020-2-1) and H_2_S (A146-1-1) assay kits (Jiancheng Bio, Nanjing, China); NSE ELISA kit (MM-0069R2, Meimian Bio, Yancheng, China); TTC (D06IS231825) and Evans Blue (O28HS199250, Yuanye Biotech, Shanghai, China); DAPI mounting medium (P0131, Beyotime, Shanghai, China); anti-CD31 (AF3628) and anti-VEGFR2 (AF644, R&D Systems, Minneapolis, MN, USA); anti-CBS (DF6410), ZO-1 (AF5145) and Claudin 5 (AF5216, Affinity Biosciences, Liyang, China); and donkey serum (G1217) and secondary antibody (GB25404, Servicebio, Wuhan, China).

### 2.2. Experimental Animals

In this study, a total of 260 wild-type (WT) adult male Sprague-Dawley (SD) rats (6–7 weeks old, 230–250 g) purchased from the Experimental Animal Center of Anhui Medical University (License No.: SCXK[Wan]2022-001). Of these, 226 rats survived the surgical procedures. Animals were maintained under specific pathogen-free (SPF) conditions in a climate-controlled room (12 h light/dark cycle, 50 ± 5% humidity, 21–25 °C) with ad libitum access to food and water. After 1-week acclimatization, experiments were conducted in accordance with the Guidelines for Animal Care and Use Committee of Anhui Medical University (Approval No.: LLSC20211062). All procedures were designed to minimize animal suffering and reduce the number of animals used.

### 2.3. MCAO/R Models

All surgical procedures were conducted following 12 h fasting with free access to water. CIRI was induced by modified transient middle cerebral artery occlusion [23]. After anesthesia with sodium pentobarbital (2%, 30 mg/kg i.p.), rats were positioned supine on a heating pad (37 °C). Following cervical hair removal and disinfection, a midline incision exposed the left common, external, and internal carotid arteries. The external carotid artery was permanently ligated, while the internal and common carotid arteries were temporarily clamped. A silicon-coated filament was inserted through an arteriotomy and advanced into the internal carotid artery (~18 mm) until resistance indicated occlusion. After 90 min of ischemia, filament withdrawal-initiated reperfusion was carried out. Rats were evaluated 24 h post-recovery using the Zea-Longa score; scores of 0 or 4 led to exclusion.

### 2.4. Experimental Protocol

In the first phase of this study, rats were randomly allocated five groups: sham group (sham), MCAO/R group (MCAO/R), AOAA group (AOAA), L-Cys group (L-Cys), and L-Cys + AOAA group (L-Cys + AOAA). This phase aimed at determining whether CBS-derived H_2_S confers protective effects against CIRI in rats.

In the second phase, animals were randomly assigned to six groups: sham group (sham), MCAO/R group (MCAO/R), VEGF_164_ group (VEGF_164_), L-Cys group (L-Cys), SU5416 group (SU5416), and L-Cys + SU5416 group (L-Cys + SU5416). This stage was designed to examine the role of VEGFR_2_ signaling in L-Cys-mediated neuroprotection.

All drugs were prepared in sterile phosphate-buffered saline (PBS). The working concentration of L-Cys solution was 100 mM [24]. Determine the optimal dose of L-Cys through preliminary experiments (Supplementary Figure S2). Vascular endothelial growth factor_164_ (VEGF_164_, 1 μg/kg), known for its potent pro-angiogenic activity, served as the positive control. After the first reperfusion, L-Cys and VEGF_164_ were administered intracerebroventricularly via an indwelling cannula. AOAA (5 mg/kg) or SU5416 (4 mg/kg) was delivered via intraperitoneal injection 30 min prior to L-Cys administration [25]. The sham and MCAO/R groups received an equivalent volume of sterile phosphate-buffered saline (PBS). Treatments were administered once a day for 14 consecutive days. Supplementary Figure S1A illustrates the overall flowchart of the study.

### 2.5. Intracerebroventricular (I.C.V.) Injection

Rats underwent stereotaxic implantation of an intracerebroventricular cannulation system targeting the lateral ventricles [26]. Under anesthesia, animals were positioned prone in a stereotaxic frame. Following scalp incision and skull exposure, bregma and lambda were identified after cleansing with 3% H_2_O_2_. A burr hole was drilled at coordinates relative to bregma (1.0 mm posterior, 1.5 mm lateral, 4.0 mm ventral) for ventricular access. The cannula was then implanted upon observation of clear cerebrospinal fluid effusion and fixed with dental cement, as shown in Supplementary Figure S1B. Post-surgical recovery allowed 3 days with daily antibiotic coverage. For drug delivery, the inner cannula was replaced with an injection assembly for controlled infusion before resealing.

### 2.6. Modified Neurological Severity Scores (mNSS)

Neurological assessment (Supplementary Table S1) was conducted by investigators blinded to experimental groups using the mNSS [27] at four predetermined timepoints (1, 3, 7, and 14 days post-reperfusion).

### 2.7. Cerebral Infarct Volume Quantification

Anesthetized rats underwent retro-orbital blood collection, the obtained samples were transferred to 1.5 mL microcentrifuge tubes and stored for subsequent analysis. Following blood collection, rats were euthanized. Whole brains were immediately dissected and flash-frozen at −20 °C for 15–20 min. The olfactory bulbs and brainstem were excised, and the remaining brain tissue was sectioned coronally into five 2 mm thick slices. These were immersed in pre-warmed (37 °C) 2% TTC solution and incubated at 37 °C in a light-protected incubator for 30 min. To ensure uniform staining, slices were turned every 10 min during incubation. TTC-stained sections were fixed in 4% paraformaldehyde (PFA) for 20 min, mounted on black boards, and imaged. Infarct volume quantification was performed using ImageJ software (version 1.54, NIH, MD, USA).

Infarct rate (%)=infarct volume/total volume×100%

### 2.8. Hematoxylin–Eosin Staining and Nissl Staining

Anesthetized rats underwent transcardial perfusion with PBS, followed by 4% PFA, intact brains were extracted and post-fixed in 4% PFA at 4 °C for 24 h. Samples were dehydrated and embedded in paraffin. Paraffin-embedded samples were sectioned coronally (4 μm). Sections were routinely deparaffinized, hydrated, and washed in distilled water, stained with hematoxylin–eosin or toluidine blue, and sealed with clear neutral resin. Tissue sections were collected and analyzed using the intelligent digital slide scanner (3D HISTECH, Budapest, BUD, Hungary) to evaluate pathological lesions. The severity of tissue damage was scored according to the criteria outlined in Supplementary Table S2: 0 points for normal, 1 point for mild/minor lesions, 2 points for moderate lesions, and 3 points for severe lesions [28].

### 2.9. Enzyme-Linked Immunosorbent Assay

We quantified the levels of LDH, NSE, and H_2_S in serum, brain tissue, and cellular samples using different types of ELISA kits. All ELISA procedures were strictly performed according to the manufacturer’s protocols.

### 2.10. Brain Fluid Content and Evans Blue Leakage Measurement

Rats were anesthetized and euthanized, followed by brain extraction and image acquisition. The ipsilateral (ischemic) hemisphere was immediately weighed to determine wet weight (WW), then dried at 100 °C for 24 h to obtain dry weight (DW).

Brain fluid content (%)=[(WW−DW)/WW]×100%

Blood–brain barrier (BBB) permeability was assessed using the Evans blue (EB) extravasation assay. After anesthetizing the rats, EB (2%, 4 mL/kg) was administered via the femoral vein and allowed to circulate for 3 h. Following transcardial perfusion with PBS, the brain was harvested. The brain tissue was homogenized in PBS, incubated in trichloroacetic acid at 37 °C for 24 h, and centrifuged at 3000 rpm for 20 min. The supernatant was collected, and its absorbance was measured at 620 nm using a microplate reader. The EB concentration in the samples was calculated based on a standard curve.

### 2.11. Immunofluorescence Staining

Tissue sections underwent baking, dewaxing, and rehydration before antigen retrieval in citrate-EDTA buffer. After three PBS washes, endogenous peroxidase was quenched with 3% H_2_O_2_ (10 min, RT). Following additional PBS washes, sections were permeabilized with Triton X-100 (20 min) and blocked with serum (1 h). Primary antibodies were applied overnight at 4 °C, followed by PBS washes and incubation with fluorophore-conjugated secondary antibodies (1 h, RT). After removing unbound antibodies, nuclei were counterstained with DAPI. Slides were mounted with anti-fade medium and imaged by fluorescence microscopy, with whole-slide digitization performed using intelligent digital slide scanner.

### 2.12. Morris Water Maze Test

Morris water maze (MWM) testing assessed rat learning and memory. The apparatus consisted of a circular pool (200 cm diameter, 50 cm height) filled with opaque water (21 cm depth, 25 ± 0.5 °C) and a hidden platform (10 cm diameter, 1 cm submerged) in the southwest (SW) quadrant. Escape latency (time to locate platform) was recorded over four training days, with rats released sequentially from SW, NW, NE, and SE quadrants (90 s trials; guided to platform if unsuccessful). On day 5, the platform was removed for a 120 s probe test, recording swim path, distance, SW quadrant crossings, and time spent in the target quadrant.

### 2.13. Open Field Test

The open field test (OFT) assessed locomotor activity and exploration behavior in rats. The arena (100 × 100 × 40 cm) was divided into central and peripheral zones. After 1 h habituation, each rat was placed in the center and allowed to explore freely for 5 min. Movement trajectory, total distance traveled, central zone entries, and rearing events were recorded. Between trials, the arena was cleaned with 75% ethanol to eliminate odor cues.

### 2.14. Cylinder Test

The cylinder test assessed post-stroke forelimb asymmetry in rats. Animals were placed in a transparent cylinder (20 cm diameter × 30 cm height) for 5 min. Forelimb use during wall exploration was scored when (1) standing fully upright while independently contacting walls during weight shift, or (2) performing alternating stepping movements with both forelimbs while standing. Recordings included ipsilateral forelimb contacts (I), contralateral forelimb contacts (C), and bilateral contacts (B).

Asymmetry score = [I/(I+C+B)]×100%.

### 2.15. Corner Test

Corner test evaluated sensorimotor asymmetry in rats. The apparatus consisted of two boards joined at a 30° angle. Rats were placed facing the corner, where simultaneous whisker stimulation on both sides triggered rearing and a 180° turn. Intact animals turned randomly left/right, whereas unilateral brain injury induced preferential turning toward the impaired side. Each rat underwent 10 trials (1 min intervals), with turns recorded only when rearing occurred. Trials without hindlimb standing were excluded.

Laterality index =(right turns/total turns).

### 2.16. Modified Adhesive Removal Test

Modified adhesive removal test evaluated sensorimotor integration by assessing forelimb sensitivity. A 3 × 1 cm paper sleeve was fitted snugly around each forepaw (allowing partial digit exposure but preventing self-removal). Normal rats vigorously removed the sleeve using their mouth or contralateral paw, whereas stroke-impaired rats showed delayed responses. Two timed measures were recorded: (1) 30 s observation period from trial initiation, and (2) latency-to-removal upon first removal attempt. Each forelimb was tested separately with three trials, averaged for analysis.

### 2.17. Western Blot

After sample processing, total protein was extracted. Protein concentration was determined using a BCA assay kit, followed by denaturation of the quantified protein samples. The proteins were then separated by sodium dodecyl sulfate–polyacrylamide gel electrophoresis (SDS-PAGE) and transferred onto a polyvinylidene difluoride (PVDF) membrane. The membrane was blocked with a blocking solution to minimize nonspecific binding and subsequently incubated with primary antibodies at 4 °C overnight. After three washes with TBST, the membrane was incubated with the corresponding secondary antibody at room temperature for 2 h. Following three additional TBST washes, enhanced chemiluminescence (ECL) reagent was applied. Protein expression levels of target bands were evaluated using an Amersham Imager 600 gel documentation system (General Electric, Boston, MA, USA). Image grayscale values were quantified and analyzed using ImageJ software. The relative expression of target proteins was determined by the ratio of their grayscale values to that of β-actin.

### 2.18. Statistical Analysis

Statistical analyses were performed using GraphPad Prism 9.5.1 (GraphPad Software, San Diego, CA, USA). Data are presented as mean ± SD after normality assessment by Shapiro–Wilk test. Parametric data were analyzed by one-way ANOVA with Tukey’s post hoc test or Student’s *t*-test; non-parametric data by Kruskal–Wallis with Dunn’s post hoc test or Mann–Whitney U test. Statistical significance was set at *p* < 0.05.

## 3. Results

### 3.1. Effect of L-Cys on Neurological Deficit Scores and Cerebral Infarction Volume in MCAO/R Rats

**Figure 1:**
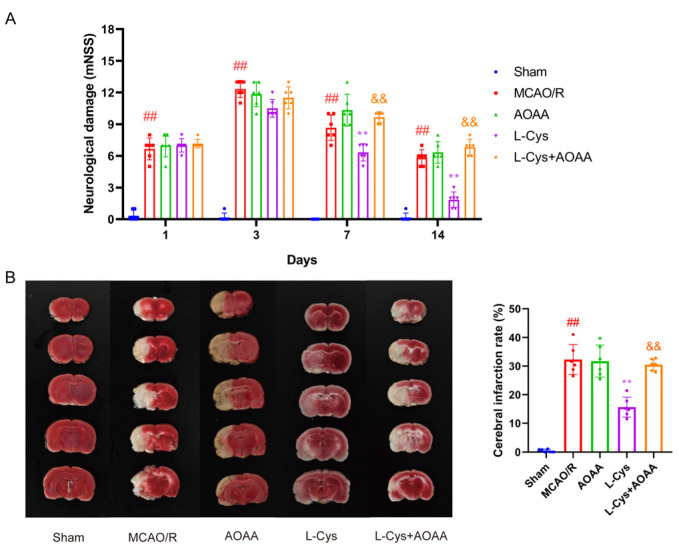
L-Cys reduced neurological deficit scores and cerebral infarction in MCAO/R rats. (**A**) Modified neurological severity score (*n* = 6). (**B**) TTC staining of brain and quantitative analysis (*n* = 6). AOAA: 5 mg/kg; L-Cys: 1.2 mg/kg. Experimental data are represented as mean ± SD. ^##^ *p* < 0.01 vs. the sham group; ** *p* < 0.01 vs. the MCAO/R group; ^&&^ *p* < 0.01 vs. the L-Cys group.

To evaluate the neuroprotective efficacy of L-Cys against CIRI, we used the established MCAO/R model in rats. As shown in Figure 1A, the neurological deficit scores were remarkably increased at 1, 3, 7, and 14 days after MCAO/R modeling compared to the sham group (*p* < 0.01). Compared with the MCAO/R group, L-Cys-treated group significantly reduced neurological deficits at 7 and 14 days, with the most pronounced effect observed at 14 days (*p* < 0.01). Consistent with the functional outcomes, through TTC staining in Figure 1B, the sham group showed no evidence of infarct, whereas the MCAO/R group exhibited a substantial increase in infarct volume (*p* < 0.01). Notably, L-Cys administration for 14 d significantly reduced cerebral infarct in MCAO/R rats (*p* < 0.01). Compared with the MCAO/R group, CBS inhibitor AOAA had no obvious effect on the cerebral infarct volume or neurological deficit. But, compared with the L-Cys group, the combination of L-Cys and AOAA significantly abolished the effect of L-Cys on both cerebral infarct volume and neurological deficit (*p* < 0.01). These results indicated that the neuroprotection conferred by L-Cys may be CBS-dependent.

### 3.2. Effect of L-Cys on MCAO/R-Induced Increases in Serum LDH and NSE Levels as Well as Cerebral Pathological Change

**Figure 2:**
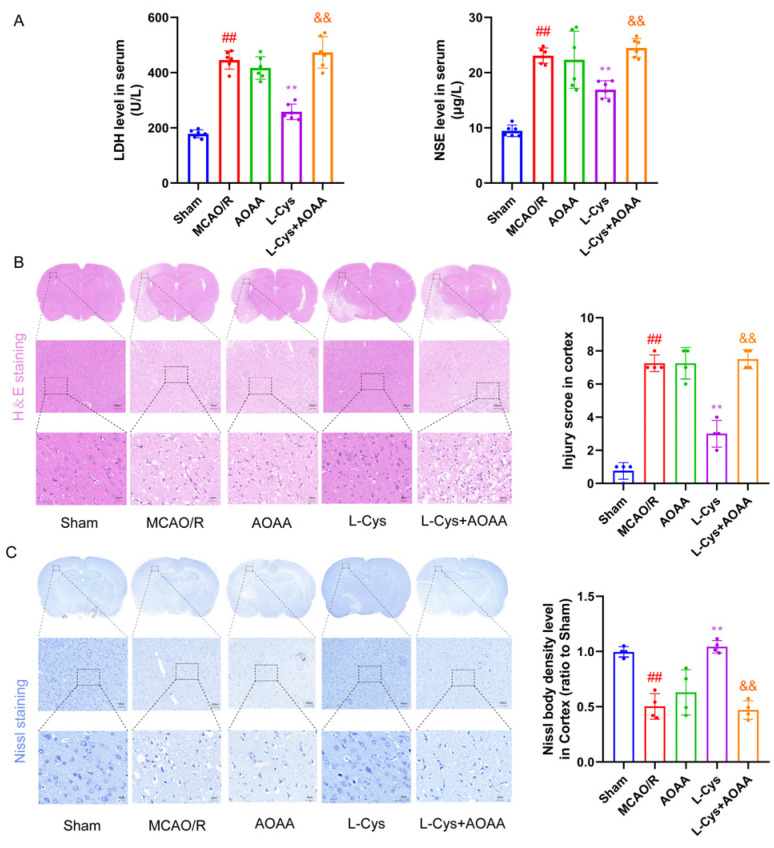
L-Cys ameliorated MCAO/R-induced increases in serum LDH and NSE levels as well as cerebral pathological change. (**A**) Levels of LDH and NSE in serum (*n* = 6). (**B**) Representative HE staining of the cortex in all groups and injury score (*n* = 4, scale bar = 25 and 100 μm). (**C**) Representative Nissl staining of the cortex in all groups and quantitative analysis of Nissl body density (*n* = 4, scale bar = 25 and 100 μm). AOAA: 5 mg/kg; L-Cys: 1.2 mg/kg. Experimental data are represented as mean ± SD. *^##^ p* < 0.01 vs. the sham group; ** *p* < 0.01 vs. the MCAO/R group; ^&&^ *p* < 0.01 vs. the L-Cys group.

To further assess neuronal injury, serum concentrations of LDH and NSE were measured. As shown in Figure 2A, both biomarkers were significantly increased in MCAO/R rats compared to the sham group (*p* < 0.01); this increase was markedly attenuated by L-Cys treatment (*p* < 0.01), an effect that was fully reversed upon co-administration with the CBS inhibitor AOAA (*p* < 0.01).

To further validate whether L-Cys mitigates CIRI at the histopathological level, we examined morphological alterations in cortical neurons of rats using H-E staining and Nissl staining. H-E staining is shown in Figure 2B. Cortical neurons in the sham group exhibited intact structure, orderly arrangement, and no evidence of vacuolar degeneration or inflammatory infiltration. In contrast, the MCAO/R group displayed extensive liquefactive necrosis, numerous eosinophilic red neurons, and substantial inflammatory cell infiltration (*p* < 0.01). These pathological alterations were markedly attenuated by L-Cys treatment (*p* < 0.01). Nissl staining in Figure 2C further confirmed severe neuronal damage following CIRI. Neurons in the MCAO/R group exhibited shrunken cell bodies and a significant decrease in Nissl-positive cells relative to the sham group (*p* < 0.01). Conversely, L-Cys administration notably increased the density of Nissl-stained neurons in the peri-infarct cortex, suggesting improved neuronal survival and structural integrity (*p* < 0.01). Importantly, the histopathological improvements mediated by L-Cys were abolished by CBS inhibition, as demonstrated by comparable staining patterns among the MCAO/R, AOAA, and L-Cys + AOAA groups.

### 3.3. Effect of L-Cys on MCAO/R-Induced Cerebral Edema and BBB Disruption

**Figure 3:**
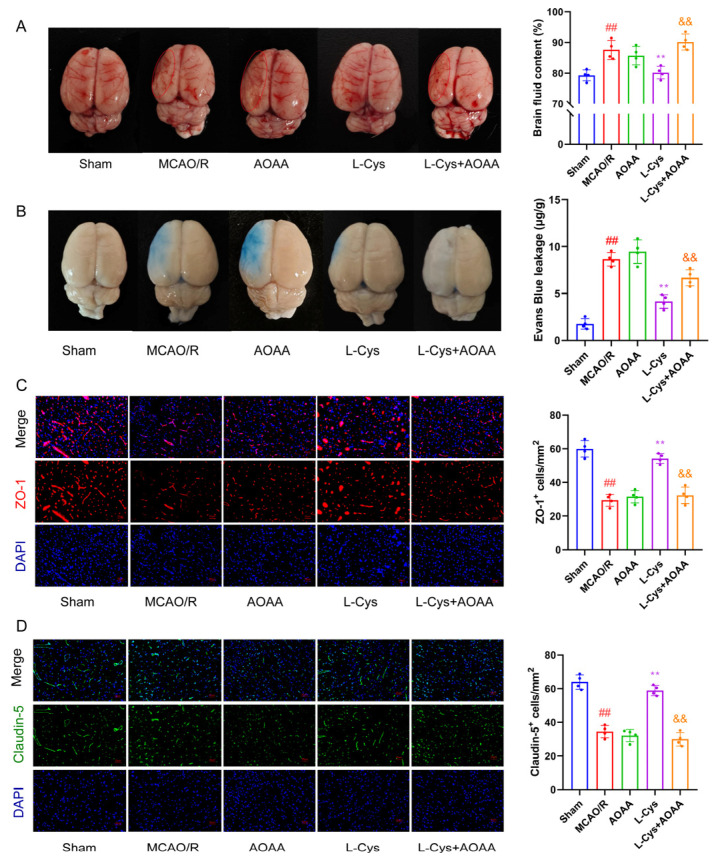
L-Cys ameliorated cerebral edema and BBB disruption in MCAO/R rats. (**A**) Representative image of cerebral edema and quantitative analysis (*n* = 4). (**B**) Representative image of EB leakage and quantitative analysis (*n* = 4). (**C**,**D**) ZO-1 and claudin-5 immunofluorescence staining of brain sections and quantitative analysis (*n* = 4, scale bar = 25 μm). AOAA: 5 mg/kg; L-Cys: 1.2 mg/kg. Experimental data are represented as mean ± SD. *^##^ p* < 0.01 vs. the sham group; ** *p* < 0.01 vs. the MCAO/R group; ^&&^ *p* < 0.01 vs. the L-Cys group.

We observed significant liquefactive necrosis in rat brain tissue 14 days after CIRI, prompting us to measure brain fluid content. As shown in Figure 3A, compared with the sham group, the MCAO/R group had significantly increased brain fluid, demonstrating cerebral edema (*p* < 0.01). L-Cys administration effectively reduced edema formation (*p* < 0.01), while concurrent AOAA treatment completely abolished this protective action.

The rapid disruption of the BBB functional and structural integrity constitutes a core component of CIRI. We assessed BBB stability through EB extravasation. As illustrated in Figure 3B, the sham group showed no significant dye leakage, whereas the MCAO/R group exhibited substantial extravasation, indicating BBB disruption. L-Cys treatment significantly reduced EB leakage, suggesting functional BBB recovery. Although the CBS inhibitor AOAA alone did not exacerbate BBB damage in MCAO/R rats, it completely abolished the protective effect of L-Cys on BBB integrity. We evaluated the expression of tight junction proteins ZO-1 and claudin-5 in rat brain tissue using immunofluorescence staining. As shown in Figure 3C,D, both ZO-1 and claudin-5 were significantly downregulated in the MCAO/R group compared with the sham group. L-Cys treatment significantly upregulated the expression of these proteins, while co-administration of L-Cys with AOAA substantially inhibited this effect. In summary, L-Cys improves BBB stability in MCAO/R rats by restoring the expression of tight junction proteins ZO-1 and claudin-5.

### 3.4. Effect of L-Cys on the Cognitive Impairment in MCAO/R Rats

**Figure 4:**
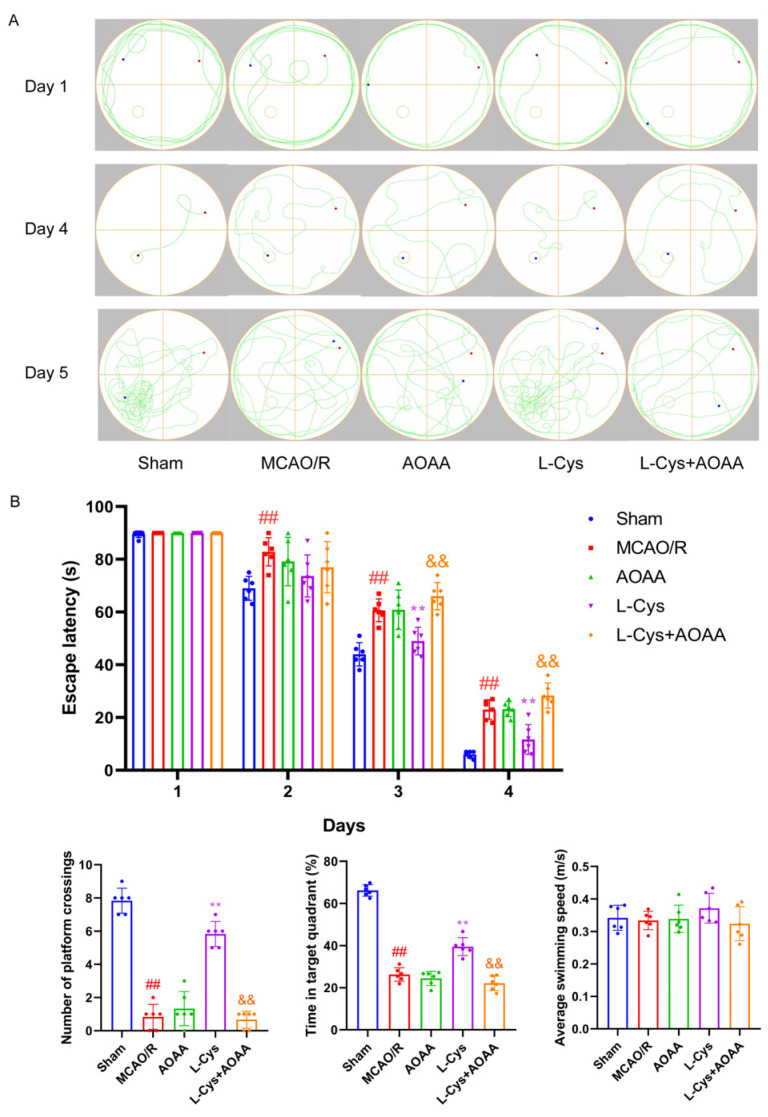
**L**-Cys improved cognitive impairment in MCAO/R rats. (**A**) Representative swimming tracks of mice from day 1, 4 and 5. The red dot is the starting point and the blue dot is the end point. (**B**) Escape latency, numbers of platform crossing, time spent in the target quadrant and average swimming speed (*n* = 6). AOAA: 5 mg/kg; L-Cys: 1.2 mg/kg. Experimental data are represented as mean ± SD. *^##^ p* < 0.01 vs. the sham group; ** *p* < 0.01 vs. the MCAO/R group; ^&&^ *p* < 0.01 vs. the L-Cys group.

The MWM test was conducted to assess the effects of L-Cys on spatial learning and memory in rats following CIRI. As illustrated in Figure 4, during the acquisition phase (days 1–4), no significant intergroup differences in escape latency were observed on day 1. As training progressed, all groups exhibited a progressive decrease in escape latency, reflecting normal learning processes. By day 4, however, MCAO/R rats displayed significantly longer escape latencies compared to the sham group (*p* < 0.01), indicating learning and memory deficits. In contrast, L-Cys treatments significantly shortened escape latency, suggesting improved cognitive recovery (*p* < 0.01). Critically, the beneficial effect of L-Cys was abolished upon co-administration with the AOAA, underscoring the dependence on CBS. During the probe trial on day 5 (with the platform removed), animals treated with L-Cys exhibited a significantly greater number of platform crossings and prolonged time spent in the target quadrant compared to the MCAO/R group (*p* < 0.01), further supporting functional cognitive restoration. Importantly, no differences in swimming speed were observed across groups, confirming that motor performance did not confound the cognitive findings.

### 3.5. Effect of L-Cys on Behavioral Deficits in MCAO/R Rats

**Figure 5:**
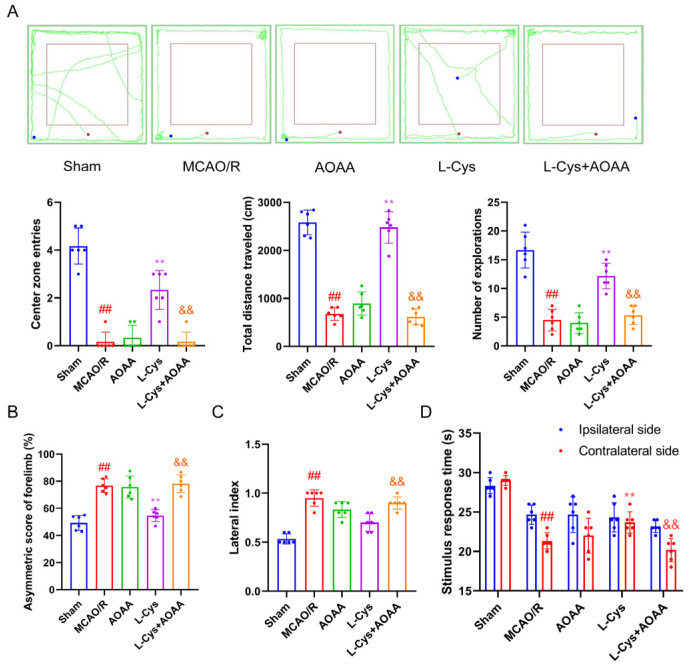
L-Cys improved behavioral deficits in MCAO/R rats. (**A**) Open-field test (*n* = 6). The red dot is the starting point and the blue dot is the end point. (**B**) Cylinder test (*n* = 6). (**C**) Corner test (*n* = 6). (**D**) Adhesive removal test (*n* = 6). AOAA: 5 mg/kg; L-Cys: 1.2 mg/kg. Experimental data are represented as mean ± SD. *^##^ p* < 0.01 vs. the sham group; ** *p* < 0.01 vs. the MCAO/R group; ^&&^ *p* < 0.01 vs. the L-Cys group.

Behavioral evaluation plays a crucial role in assessing functional restoration in experimental stroke. We utilized an integrated behavioral paradigm comprising open field, cylinder, corner, and adhesive removal tests to systematically analyze sensorimotor and exploratory impairments induced by CIRI. As illustrated in Figure 5A, MCAO/R animals showed markedly suppressed exploratory activity in the open field test relative to sham controls, manifesting as diminished center zone entries, shortened travel path, and reduced rearing frequency. Figure 5B displays evident forelimb asymmetry in the cylinder test, where ischemic rats preferentially used their ipsilateral limb during vertical exploration. Correspondingly, Figure 5C exhibits distinct turning preference in the corner test, indicating compromised lateralized sensorimotor coordination. Additionally, delayed response to tactile stimulation was detected in the adhesive removal test (Figure 5D), suggesting impaired sensorimotor integration. Together, these findings demonstrate widespread functional deficits resulting from ischemic insult. Administration of L-Cys substantially mitigated these impairments; nevertheless, co-treatment with the CBS inhibitor AOAA largely reversed the therapeutic benefits in functional recovery.

### 3.6. Effect of L-Cys on CBS and VEGFR_2_ Expressions and Endogenous H_2_S Production in MCAO/R Rat Brain

**Figure 6:**
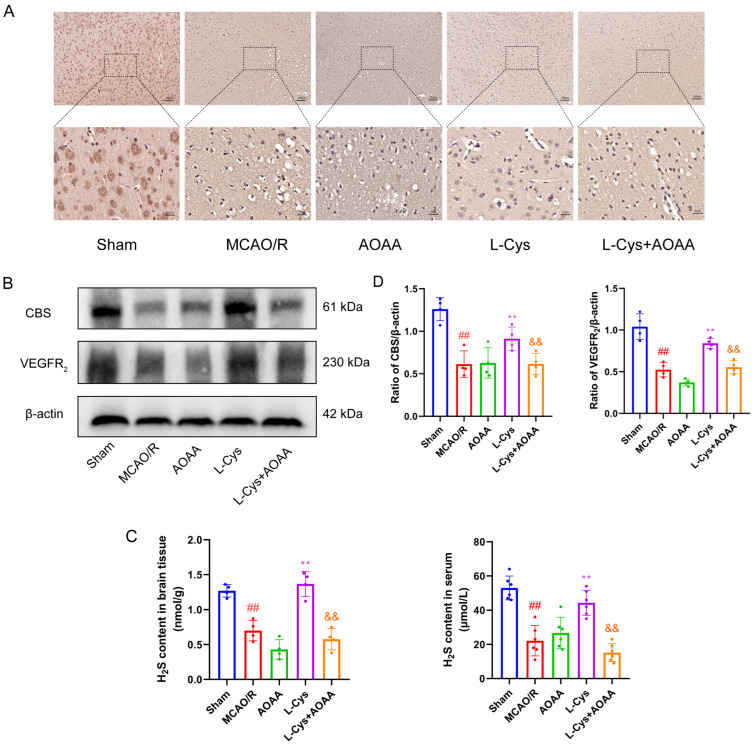
L-Cys upregulated CBS and VEGFR_2_ expression and endogenous H_2_S production in MCAO/R rats. (**A**) Immunohistochemical staining of brain sections for CBS (*n* = 4, scale bar = 25 μm and 100 μm ). (**B**) Western blot analysis of CBS and VEGFR_2_ in brain (*n* = 4). (**C**,**D**) Determination of H_2_S content in brain tissue and serum (*n* = 4 or 6). AOAA: 5 mg/kg; L-Cys: 1.2 mg/kg. Experimental data are represented as mean ± SD. *^##^ p* < 0.01 vs. the sham group; ** *p* < 0.01 vs. the MCAO/R group; ^&&^ *p* < 0.01 vs. the L-Cys group.

CBS is indispensable for the endogenous synthesis of H_2_S within the brain. In this study, we sought to determine whether the neuroprotection conferred by L-Cys in MCAO/R rats involves the modulation of CBS expression and subsequent H_2_S production. We employed immunohistochemical staining to observe changes in cortical CBS-positive neurons. As shown in Figure 6A, compared with the sham group, the number of CBS-positive neurons in the cerebral cortex of MCAO/R rats was significantly reduced, whereas the L-Cys group exhibited a significant increase. To further validate alterations in CBS expression, we analyzed CBS protein levels in cortical tissue via WB (Figure 6B). Consistent with immunohistochemical findings, CBS protein expression was significantly downregulated in the MCAO/R group relative to the sham group, while L-Cys treatment markedly reversed this downregulation trend. Additionally, we measured H_2_S levels in brain tissue and serum (Figure 6C). Results showed that H_2_S levels in both brain tissue and serum of MCAO/R rats were significantly lower than those in the sham group. Following L-Cys treatment, H_2_S levels in both brain tissue and serum significantly rebounded. Administration of L-Cys effectively increased CBS expression and H_2_S concentrations in MCAO/R rats, but this increase was abolished by AOAA, indicating that L-Cys may recover H_2_S production via the CBS in the cerebrum in the MCAO/R rat. Remarkably, L-Cys treatment also resulted in a significant upregulation of VEGFR_2_ protein expression in the peri-infarct cortex, which was significantly attenuated by AOAA, suggesting a potential interaction between H_2_S signaling and VEGFR_2_ expression in rat brain.

### 3.7. Effect of VEGFR_2_ Inhibitor SU5416 on L-Cys Reducing the Neurological Deficit and Cerebral Infarction in MCAO/R Rats

**Figure 7:**
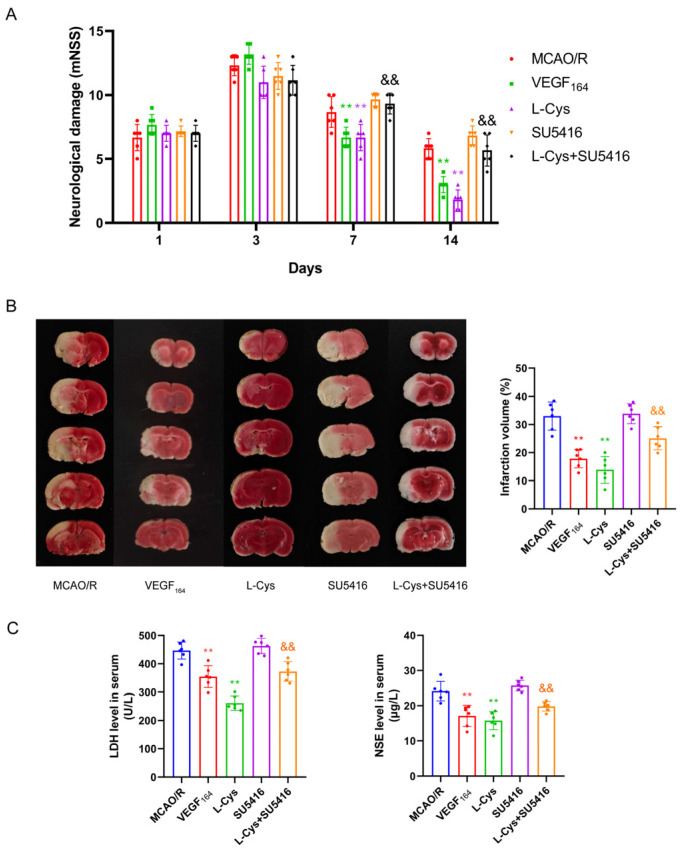
SU5416 inhibits L-Cys-induced therapeutic benefits in MCAO/R rats. (**A**) Modified neurological severity score (*n* = 6). (**B**) TTC staining of brain and quantitative analysis (*n* = 6). (**C**) Levels of LDH and NSE in serum (*n* = 6). VEGF_164_: 1 μg/kg; SU5416: 4 mg/kg; L-Cys: 1.2 mg/kg. Experimental data are represented as mean ± SD. ** *p* < 0.01 vs. the MCAO/R group; ^&&^ *p* < 0.01 vs. the L-Cysteine group.

To determine whether the neuroprotection offered by L-Cys in MCAO/R rats involves VEGFR_2_ signaling, we assessed the influence of VEGF_164_ and the selective VEGFR_2_ inhibitor SU5416 on L-Cys reducing the neurological deficit and cerebral infarction. As illustrated in Figure 7, administration of VEGF_164_ for 14 days resulted in significant improvements in neurological function and a reduction in infarct size. SU5416 treatment alone did not significantly affect neurological deficits or cerebral infarct volume in MCAO/R rats. However, co-administration of L-Cys with SU5416 resulted in partial but significant reversal of its beneficial effects. These findings suggest that activation of the VEGFR_2_ signaling pathway likely mediates the neuroprotective mechanisms of L-Cys. VEGF_164_ treatment similarly reduced serum levels of LDH and NSE in rats, paralleling the effects of L-Cys. This reduction was partially but significantly reversed upon VEGFR_2_ inhibition. These findings further support the conclusion that activation of the VEGFR_2_ signaling pathway likely mediates the neuroprotective mechanism of L-Cys. However, the SU5416 inhibition was incomplete, indicating that the protective effect of L-Cys involves non-VEGF-dependent pathways.

### 3.8. Effects of L-Cys on Cerebral Angiogenesis and Cerebral Blood Flux in MCAO/R Rats

**Figure 8:**
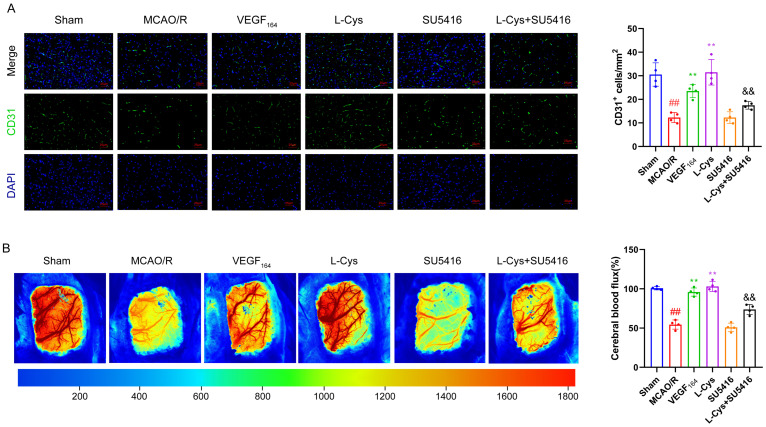
L-Cys promoted angiogenesis and CBF recovery. (**A**) CD31 immunofluorescence staining of brain sections and quantitative analysis (*n* = 4, scale bar = 25 μm). (**B**) Representative laser speckle contrast images of ischemic hemisphere and quantitative analysis of cerebral blood flux (*n* = 4). VEGF_164_: 1 μg/kg; SU5416: 4 mg/kg; L-Cys: 1.2 mg/kg. Experimental data are represented as mean ± SD. *^##^ p* < 0.01 vs. the sham group; ** *p* < 0.01 vs. the MCAO/R group; ^&&^ *p* < 0.01 vs. the L-Cys group.

To determine whether L-Cys promotes post-ischemic cerebrovascular regeneration, we evaluated microvascular density via immunofluorescence staining of the endothelial marker CD31. As shown in Figure 8, both VEGF_164_ and L-Cys significantly increased the CD31-positive cell density compared to the MCAO/R group, indicating enhanced cerebral angiogenesis. Consistent with these findings, laser speckle contrast imaging revealed a significant decrease in cerebral blood flux (CBF) following MCAO/R injury, which was substantially restored by either VEGF_164_ or L-Cys treatment. Notably, the pro-angiogenic and CBF-improving effects of L-Cysteine were markedly attenuated by co-administration of the VEGFR_2_ inhibitor SU5416. These results demonstrate that activation of the VEGFR_2_ signaling pathway contributes critically to L-Cys-mediated angiogenesis and cerebral perfusion recovery after ischemic insult.

## 4. Discussion

Accumulating evidence indicates that endogenous H_2_S exerts diverse biological functions in mammalian cells at physiologically relevant concentrations [7,29]. H_2_S interacts with multiple signaling molecules, modulates signal transduction pathways, and plays critical roles in various pathological conditions including myocardial infarction [30], heart failure [31], myocardial ischemia–reperfusion injury [32] and neurodegenerative disorders [33]. Given these pleiotropic effects, H_2_S-based therapeutics have emerged as a promising strategy for ischemic stroke treatment. However, the clinical translation of exogenous H_2_S delivery remains challenging due to limitations in stability, solubility, target specificity, and short half-lives associated with current administration methods such as direct inhalation or H_2_S-releasing donors [34]. In contrast, neuronal H_2_S, primarily generated by CBS in the brain, plays crucial roles in neurotransmission and neuroprotection [35].

In this study, MCAO/R-induced injury in rats resulted in significant downregulation of CBS expression in ischemic brain tissue, accompanied by markedly reduced H_2_S levels in both the brain tissue and serum. L-Cys administration effectively restored CBS expression and H_2_S levels, thereby attenuating CIRI as evidenced by reduced neurological deficits (mNSS), decreased cerebral infarct volume, and ameliorated histopathological damage, cerebral edema, and BBB disruption. However, it is important to acknowledge that L-Cys serves as a precursor for multiple bioactive metabolites via the CBS pathway, each potentially playing distinct roles in cerebral ischemia. L-Cystathionine, the direct product of the CBS-catalyzed condensation of L-Cys and homocysteine, is clinically recognized as an independent risk marker for cardiovascular disease, with elevated circulating levels positively correlating with oxidative damage and endothelial dysfunction [36,37]. Cystathionine is not a metabolic endpoint; it can be reversibly converted back to L-Cys by CSE, linking it directly to the regeneration of the substrate pool [12]. Furthermore, downstream metabolites of L-Cys, such as glutathione and taurine, are well-established antioxidants and cytoprotective agents that may contribute to long-term recovery and homeostasis maintenance after ischemic injury [38]. CBS is primarily expressed in the central nervous system, whereas CSE is mainly distributed in the cardiovascular system. Our previous study demonstrated that CSE in cerebral vascular endothelial cells exerts neuroprotective effects independent of CBS, suggesting that CSE may serve a compensatory function when CBS is inhibited [39]. And in neuroglial cells subjected to hypoxia/reoxygenation (H/R) injury, we observed that CBS expression was significantly decreased, whereas 3-MST expression showed no significant change. This suggests that 3-MST may be less responsive to ischemic stress compared to CBS, and that compensatory H_2_S production from 3-MST during CIRI may be limited [40]. While the metabolic complexity of the CBS pathway precludes exclusive attribution to H_2_S, our findings strongly demonstrate that neuronal CBS-derived H_2_S serves as an important reparative factor following IS. The future research use of specific H_2_S scavengers will further strengthen this causal inference.

In IS, increased microvascular density in peri-infarct regions correlates strongly with improved survival outcomes, positioning pro-angiogenic therapies as a promising regenerative strategy [41]. While tightly regulated under physiological conditions, angiogenesis is reactivated following stroke in both pre-clinical models and patients [42]. Revascularization of ischemic areas restores microcirculation, facilitating delivery of nutrients and growth factors that support neuronal survival and neural progenitor activity—constituting a critical endogenous neurorepair mechanism [43]. The role of H_2_S in angiogenesis has been extensively demonstrated; delivery of H_2_S to endothelial cells significantly promotes cell proliferation, migration and capillary tube formation [44,45]. For example, Qi et al. [46] demonstrated that enhanced stromal cell CBS-H_2_S production promotes estrogen-stimulated human endometrial angiogenesis. Consistent with the theoretical framework, our results demonstrate that CBS-derived H_2_S promotes angiogenesis in the cerebral infarct region, enhances effective cerebral reperfusion, and substantially improves neuronal survival. Furthermore, CBS-derived H_2_S restored the expression of ZO-1 and claudin-5, which is consistent with the established mechanism of H_2_S-mediated BBB protection. Li et al. demonstrated that H_2_S exerts its effects by inhibiting pro-damaging PKC isoforms (PKC-α, βI, βII, δ) whilst promoting the protective PKC-ε isoform [47]. H_2_S also inhibits MMP-9 activity, thereby preventing the degradation of tight junction proteins [48], and suppresses ERK/JNK/p38 phosphorylation, restoring ZO-1 expression [49]. This study did not directly evaluate these upstream signaling events; future research will examine the PKC isoform profile, MMP-9 activity and MAPK phosphorylation induced by CBS-derived H_2_S to conclusively establish the molecular relationships within CIRI model.

Growing evidence suggests that VEGFR_2_ may serve as a direct target of H_2_S. Tao et al. demonstrated that the H_2_S donor NaHS can cleave the disulphide bond between Cys1045 and Cys1024 in VEGFR_2_ on vascular endothelial cells, and promotes H_2_S-dependent angiogenesis in a mice Matrigel embolism model [20]. Similarly, Zhu et al. [50] reported that NaHS ameliorates primary hypertension by modulating endothelial function via VEGFR_2_ activation. Our prior work revealed that H_2_S-induced vasodilation in rat vascular smooth muscle cells is potentially mediated by VEGFR_2_ [51]; in vitro, NaHS enhances phosphorylation of VEGFR_2_ at Tyr797 and Ser799, thereby exerting pro-angiogenic effects and protecting against hypoxia–reoxygenation (H/R) injury [22], and total flavones of *Rhodiola japonica* promoted vascular formation by inducing CBS synthesis of H_2_S, which acts on VEGFR_2_ in rat cerebral vascular endothelial cells [52]. Consistent with these findings, our findings indicated that L-Cys upregulated VEGFR_2_ expression in rat brain tissue, while the protective effects of neuronal CBS-derived H_2_S are blocked by VEGFR_2_ inhibitor SU5416. In accordance with this, CBS-derived H_2_S functions as a key neuromodulator in post-ischemic cerebrovascular repair, highlighting its therapeutic potential as a target for intervention in IS.

However, VEGFR_2_ inhibitor SU5416 only partially counteracted the protective effect of L-Cys against CIRI, strongly suggesting that the protective action of L-Cys/H_2_S may involve mechanisms independent of the VEGF signaling pathway. Research has revealed that L-Cys itself serves as a potent antioxidant. Even under conditions where glutathione synthesis is inhibited, L-Cys remains effective in scavenging reactive oxygen species, protecting mitochondrial function, alleviating endoplasmic reticulum stress, restoring the mitochondrial fission–fusion dynamic equilibrium and safeguards neurons from oxidative stress damage [53]. H_2_S itself exerts a broad range of effects. It can directly inhibit apoptosis by activating the Akt and ERK1/2 signaling pathways, suppress NF-κB nuclear translocation, reduce the release of proinflammatory factors (TNF-α, IL-1β, IL-6), inhibit M1 polarization of microglia, and enhance the stability of tight junction proteins (ZO-1, Occludin) through direct sulfhydryl modification [34,54,55]. Furthermore, as gaseous signaling molecules, hydrogen sulfide and nitric oxide exhibit complex cross-regulatory interactions in ischemic brain injury. Research indicates that hydrogen sulfide can synergistically dilate cerebral blood vessels with NO by directly upregulating the expression and activity of endothelial nitric oxide synthase (eNOS), thereby improving blood supply to ischemic regions [56,57]. The synergistic interaction between H_2_S and NO enhances NO-induced angiogenesis [58]. The increase in CBF observed following L-Cys treatment in this study may be partially attributable to H_2_S’s regulatory effects on the NO pathway.

The findings of this study hold potential for clinical translation and may offer novel insights into addressing key challenges in the treatment of IS. Currently, EVT remains the most effective acute-phase intervention for stroke, but its application is constrained by a strict time window and carries risks of reperfusion injury and hemorrhagic transformation. Therefore, there is an urgent need for neuroprotective agents with vascular protective properties. Our findings provide candidate solutions for this therapeutic strategy. L-Cys, as an endogenous amino acid in the human body, possesses relatively well-established safety characteristics. This study confirms that L-Cys not only reduces infarct volume and improves neurological function but also plays a crucial role in maintaining the integrity of the BBB. Of course, our study has limitations. This study did not directly measure the phosphorylation levels of VEGFR_2_ or key downstream signaling molecules in brain tissue following L-Cys treatment; further research will be conducted to investigate the molecular mechanisms underlying the protective effects of brain-derived H_2_S, building on the findings of this study. Furthermore, we lack detection of H_2_S concentrations during cerebral ischemia, and further research is needed on the therapeutic efficacy of H_2_S at different stages of the ischemic process. In a study by Ren et al., using a whole-brain ischemia–reperfusion model in rats, H_2_S levels in the hippocampus and cortex rose significantly 12 h after reperfusion, fell markedly after 24 h, and returned to the levels observed in the sham-surgery group after 48 h, remaining stable for up to 7 days [59]. The introduction of dynamic monitoring of H_2_S concentrations may help to determine the optimal timing for L-Cys administration. In this study, we administered L-Cys (1.2 mg/kg) via ICV injection to act directly on the central nervous system (CNS). However, ICV injection is an invasive procedure and is not suitable for routine clinical treatment of patients with acute stroke. Due to differences in bioavailability and penetration into the CNS, this dosage cannot be directly translated into a systemic administration regimen. It is therefore necessary to explore alternative routes of administration. In clinical practice, IS patients often present for treatment several hours after the onset of symptoms; the therapeutic time window requires systematic evaluation, such as whether treatment remains effective 3, 6 or 12 h after ischemia. As Wu et al. noted [5], treatment of ischemic stroke should target the entire pathological process. Developing H_2_S modulators based on concentration, location, and time-dependent effects of H_2_S generation may achieve optimal therapeutic outcomes. To achieve clinical translation, efficacy must be validated in large animal models that more closely mimic clinical settings. Systematic long-term toxicological and pharmacokinetic studies are also required to determine the safe dosage range. Furthermore, given the complexity of L-Cys metabolism, developing a new generation of H_2_S-releasing drugs with enhanced targeting and stability may represent a promising avenue for further improving therapeutic efficacy and safety.

## 5. Conclusions

The present study elaborates that L-Cys attenuates CIRI by upregulating CBS expression in the ischemic penumbra, enhancing neuronal H_2_S biosynthesis, and activating VEGFR_2_ to promote post-ischemic cerebral angiogenesis. This cascade of events leads to improved cerebral reperfusion, reduced neurological deficits and infarct volume, and alleviated cerebral edema and BBB disruption. Our study not only provides scientific evidence for the future clinical application of neuronal H_2_S in cerebral microcirculatory disorders but also reveals a novel potential therapeutic target for ischemic cerebrovascular diseases.

## Data Availability

The original contributions presented in this study are included in the article/Supplementary Materials. Further inquiries can be directed to the corresponding authors.

## Supplementary Materials

The following supporting information can be downloaded at: https://www.mdpi.com/article/10.3390/cimb48040418/s1.

## Abbreviations

The following abbreviations are used in this manuscript:

IS	Ischemic stroke
CIRI	Cerebral ischemia–reperfusion injury
VEGF	Vascular endothelial growth factor
VEGF_164_	Vascular endothelial growth factor 164
VEGFR_2_	Vascular endothelial growth factor receptor-2
L-Cys	L-Cysteine
CBS	Cystathionine-β-synthase
MCAO/R	Middle cerebral artery occlusion/Reperfusion
LDH	Lactate dehydrogenase
NSE	Neuron specific enolase
HE	Hematoxylin–eosin staining
BBB	Blood–brain barrier
ZO-1	Tight junction protein 1
CD31	Platelet endothelial cell adhesion Molecule-1
CBF	Cerebral blood flux
