# Supplementary material for: Brain-Derived Cystathionine β-Synthase-Generated H2S Attenuates Cerebral Ischemia–Reperfusion Injury via VEGFR2-Mediated Angiogenesis in MCAO/R Rats
Source: Curr Issues Mol Biol. 2026 Apr 18;48(4):418. doi: 10.3390/cimb48040418 (PMC13114484; doi:10.3390/cimb48040418)
Supplement: Supplementary file 1 [file cimb-48-00418-s001.zip › cimb-4230733-supplementary.pdf]

Supplementary Information

Brain-Derived Cystathionine β-Synthase-Generated H_2S Attenuates Cerebral Ischemia–Reperfusion Injury via $VEGFR_2$-Mediated Angiogenesis in MCAO/R Rats

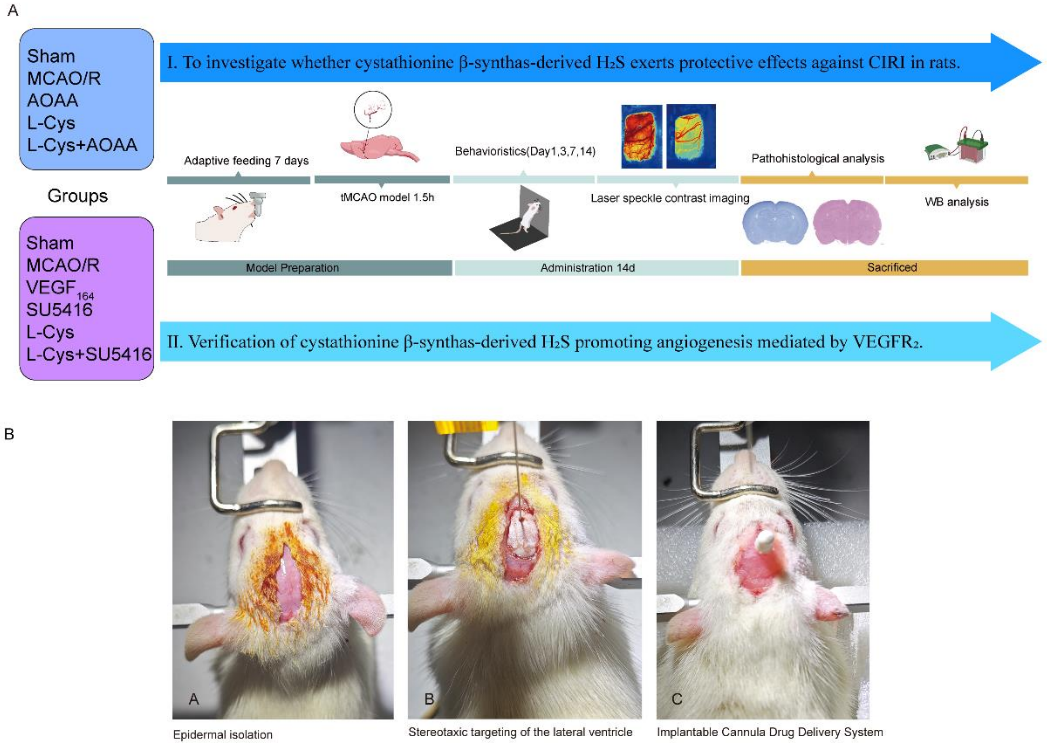

Supplementary Figure S1. Experimental protocol. (A) Schematic diagram for experimental design. (B) Intracerebroventricular injection.

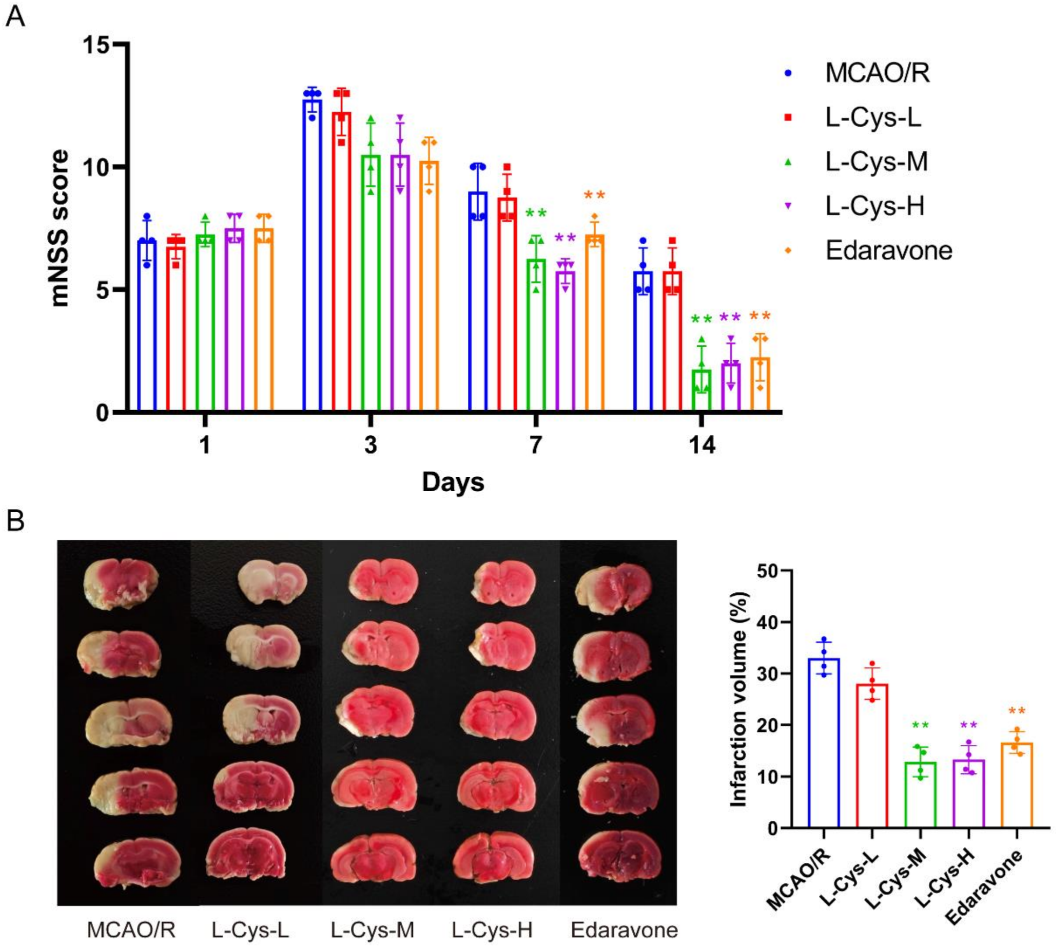

Supplementary Figure S2. Effects of different doses of L-Cys on CIRI in rats. **(A)** Modified neurological severity score (n=4). **(B)** TTC staining of brain and quantitative analysis (n=4). L-Cys-L: 0.6mg/kg; L-Cys-M: 1.2mg/kg; L-Cys-H: 2.4mg/kg; Edaravone: 5mg/kg. Experimental data are represented as mean ± SD. $^{**}p < 0.01$ vs. the MCAO/R group.

Supplementary Table S1. Modified neurological severity scores

Neurological Deficit Score	Score
**Motor Function Tests**	
Tail Suspension Test	
Forelimb Flexion	1
Hindlimb Flexion	1
Head Deviation >10° from Vertical Axis within 30s	1
Placing Rat on Floor (Normal Score = 0; Maximum Score = 3)	
Normal walking	0
Failure to walk in a straight line	1
Circling toward the paretic side	2
Falling toward the paretic side	3

**Sensory Function Tests**	
Placing Test (Visual and Tactile)	1
Proprioceptive Test (Deep Sensation)	1
Balance Beam Test (Normal Score = 0; Maximum Score = 6)	
Maintains stable posture	0
Grasps the edge of the beam	1
Hugs the beam with one limb hanging down	2
Hugs the beam with two limbs hanging down or spins on the beam	3
(>60 s)	4
Attempts to balance on the beam but falls (>40 s)	5
Attempts to balance on the beam but falls (>20 s)	6
Falls; no attempt to balance on the beam (<20 s)	
**Loss of Reflexes and Abnormal Movements**	
Auricular Reflex	1
Corneal Reflex	1
Startle Reflex	1
Seizures, Myoclonus, Dystonia	1

Supplementary Table S2. H-E staining injury score

**Score**	0	1	2	3
Liquefying necrosis of infarct	-	+	++	+++
Red neuron	-	+	++	+++
Inflammatory cell infiltration	-	+	++	+++
